# Pilot study: the impact of simulation-based pediatric basic life support training on the performance of caregivers of children with Rett syndrome

**DOI:** 10.1186/s12909-026-09158-y

**Published:** 2026-04-10

**Authors:** Seymanur Kocyigit, Ozlem Akgun-Dogan, Ozden Hatırnaz Ng, Yasemin Alanay, Feray Guven

**Affiliations:** 1 Department of Pediatrics, School of Medicine, Acibadem Mehmet Ali Aydinlar University, Istanbul, Turkey; 2 Division of Pediatric Genetics, Department of Pediatrics, School of Medicine, Acibadem Mehmet Ali Aydinlar University, Istanbul, Turkey; 3 Rare Diseases and Orphan Drugs Application and Research Center (ACURARE), Acibadem Mehmet Ali Aydinlar University, Istanbul, Turkey; 4 Department of Translational Medicine, Graduate School of Health Sciences, Acibadem Mehmet Ali Aydinlar University, Istanbul, Turkey; 5 Department of Medical Biology, School of Medicine, Acibadem Mehmet Ali Aydinlar University, Istanbul, Turkey; 6 Department of Medical Services and Techniques, Vocational School of Health Services, Acibadem Mehmet Ali Aydinlar University, Istanbul, Turkey; 7 Centre of Advanced Simulation and Education, Acibadem Mehmet Ali Aydinlar University, Istanbul, Turkey

**Keywords:** Rett syndrome, Pediatric basic life support, Critical care, Caregivers, Caregiver training, Health education, Simulation-based education, Rare diseases

## Abstract

**Background:**

Rett syndrome is a rare neurodevelopmental disorder associated with an increased risk of severe respiratory dysfunction and cardiorespiratory instability. In some individuals, particularly those with autonomic dysfunction or QT interval prolongation, life-threatening cardiac events may occur. Given the frequency of life-threatening emergencies in affected children, it is essential that primary caregivers are trained in pediatric basic life support (PBLS). This pilot study aimed to evaluate the effectiveness of simulation-based PBLS training for caregivers of children with Rett syndrome.

**Methods:**

Ten caregivers, all members of the Turkish Rett Syndrome Federation and primary caregivers of affected children, participated in a structured PBLS training program conducted in a university-based simulation center. Participants underwent pre- and post-training performance assessments using simulation scenarios based on the 2021 European Resuscitation Council (ERC) PBLS guidelines. Performance was scored using a standardized checklist, and post-training feedback was collected through a satisfaction questionnaire.

**Results:**

Pre-training scores indicated limited PBLS proficiency (mean:15.9/100). Post-training performance improved significantly (mean: 79.3/100), with notable gains across all critical PBLS steps. Questionnaire results confirmed high levels of participant satisfaction, increased self-confidence, and a perceived sense of preparedness for emergency response.

**Conclusion:**

Simulation-based PBLS training is an effective educational approach for caregivers of children with Rett syndrome, leading to substantial improvement in both skill acquisition and confidence. This initiative represents the first effort in Turkey to utilize medical simulation centers for caregiver education in the context of rare diseases, highlighting its potential for broader public health applications.

**Trial registration:**

Clinical trial number: not applicable.

**Supplementary Information:**

The online version contains supplementary material available at 10.1186/s12909-026-09158-y.

## Introduction

Rett syndrome is a rare, severe, and progressive neurodevelopmental disorder that affects multiple organ systems, predominantly in females. It is most commonly associated with pathogenic variants in the methyl-CpG-binding protein 2 (*MECP2*) gene on the X chromosome [1]. Although Rett syndrome was historically described as having an initial period of typical development, recent evidence suggests subtle abnormalities may be present from early infancy [2]. MECP2 dysfunction disrupts epigenetic regulation, leading to cumulative changes that may drive neuronal dysfunction and regression [3]. Recent systematic reviews and meta-analyses estimate the worldwide prevalence of Rett syndrome at approximately 7.1 per 100,000 females [4]. Due to its X-linked genetic basis, the condition is considerably rarer in males.

The clinical course of Rett syndrome includes a wide spectrum of complications such as motor skill regression, respiratory dysfunction, feeding difficulties, scoliosis, sleep disturbances, and epileptic seizures [1]. Several clinical features in Rett syndrome contribute to increased medical vulnerability. Epileptic seizures are common and may fluctuate in severity, and some individuals exhibit cardiac involvement such as prolonged QT interval or susceptibility to arrhythmias [5, 6]. Transient rhythm disturbances—including bradycardia or brief asystolic episodes during or after seizures—have also been reported [7]. In addition, individuals with Rett syndrome who develop epilepsy may also be at risk for sudden unexpected death in epilepsy (SUDEP). Although the exact contribution of SUDEP to mortality in Rett syndrome is not fully defined, epilepsy-related cardiorespiratory dysfunction has been suggested as a possible mechanism contributing to sudden death in this population. All of these factors may increase the risk of acute deterioration in some patients.

Although advances in supportive care have improved life expectancy, individuals with Rett syndrome remain at increased risk of premature and sudden death compared with the general population. The leading causes of mortality include cardiorespiratory complications (e.g., pneumonia, respiratory failure, and aspiration) and sudden unexpected death, while severe seizures and malnutrition are less frequent contributors. The underlying mechanisms of sudden death remain incompletely understood [8]. Annual mortality is estimated at 1.2%, with sudden unexplained death accounting for approximately 26% of cases and an additional 13% occurring in individuals with a history of severe seizures [9].

Given the high risk of life-threatening events, caregivers of children with Rett syndrome must be prepared to respond promptly and effectively in emergencies. The persistent risk of cardiopulmonary arrest may contribute to caregiver anxiety and emotional burden [10], and lack of PBLS training can lead to hesitation or delayed intervention due to fear of causing harm [11].

Simulation-based education is widely used in medical training for its ability to enhance both knowledge and practical skills [12]. By providing a safe and controlled environment, it allows learners to practice without risk and build confidence through guided feedback [13]. Previous studies have demonstrated that simulation-based BLS training for parents of high-risk infants, including those in NICUs, significantly improves emergency response performance [14].

The aim of this pilot study was to evaluate whether simulation-based PBLS training improves caregivers’ ability to recognize and respond to life-threatening emergencies that may occur in children with Rett syndrome, including apnea, seizure-related respiratory compromise, and cardiopulmonary arrest.

## Methods

This study implemented a comprehensive, simulation-based emergency training program designed specifically for caregivers of children with Rett syndrome. The program combined multiple structured components, including PBLS, seizure first aid, and airway protection strategies. In addition, caregivers received training on the management of choking events that may occur during feeding due to swallowing dysfunction frequently observed in Rett syndrome. This component followed current pediatric first-aid recommendations for airway obstruction management and the Heimlich maneuver when applicable for appropriate age groups. The training aimed to develop both theoretical understanding and practical psychomotor skills through a standardized sequence of instructional modules, scenario-based simulations, and structured debriefing.

**Fig. 1:**
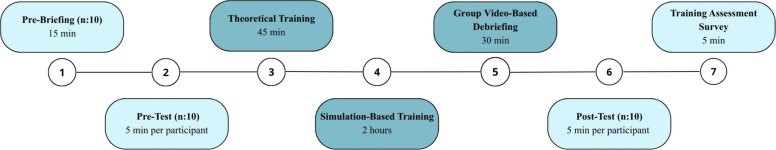
The flow-chart of the study

PBLS was selected as the primary outcome measure because it provides standardized and objectively assessable psychomotor skills suitable for pre–post comparison in a pilot design. This single-group pre–post study included ten volunteer caregivers recruited through the national Rett syndrome family association (Fig. 1).

### Eligibility criteria

Eligibility criteria were: (I) being the primary caregiver (parent or legal guardian) of a child with a confirmed Rett syndrome diagnosis; (II) ability to attend the full 1-day simulation training; and (III) voluntary participation in both pre- and post-training assessments.

All participating children had previously received a clinical diagnosis of classical Rett syndrome according to the 2010 diagnostic criteria [15]. Diagnoses were established by experienced pediatric neurologists and/or clinical geneticists at tertiary care centers in Turkey. In several cases, the diagnosis had been confirmed by more than one specialist during routine clinical follow-up. In all cases, the clinical diagnosis was further supported by molecular testing demonstrating pathogenic or likely pathogenic variants in the *MECP2* gene. Caregivers who had received previous formal pediatric basic life support training or who were not the primary daily caregiver were excluded.

### Study time frame and location

The 1-day simulation-based training program was conducted at the Acıbadem University Clinical Simulation and Advanced Training Center (CASE), Istanbul, Türkiye. All pre-training assessments, simulation activities, debriefings, and post-training evaluations were carried out in the same facility to ensure standardization of the educational environment.

The following steps were implemented sequentially to ensure comprehensive learning and performance assessment:

### Pre-briefing

A structured pre-briefing was conducted before the simulation. Facilitators outlined the learning objectives, explained the scenario flow, and oriented participants to the simulation environment and equipment, including pediatric mannequins and cardiopulmonary resuscitation (CPR) feedback devices.

Participants were informed about study procedures and provided written informed consent. A brief demographic form was completed, collecting caregiver and child characteristics, including epilepsy status, swallowing difficulties, and prior PBLS training experience.

Psychological safety was emphasized through a non-judgmental and confidential learning environment, and the Basic Assumption (“All participants are intelligent, capable, and trying their best”) was explicitly stated. This brief orientation ensured that all caregivers understood the scenario structure and felt comfortable before starting the PBLS simulation.

### Pre-training performance assessment

Before training, each participant completed a single-rescuer PBLS simulation using pediatric mannequins to reflect real-life emergency scenarios. Performance was independently assessed by two expert instructors using a 16-step checklist developed through the Delphi method and aligned with the 2021 ERC PBLS Guidelines [16]. Each item was scored as 10 (correct), 5 (partially correct), or 0 (incorrect or omitted).

Checklist items were evaluated through structured direct observation. “Checking for clear signs of life” required confirming unresponsiveness and identifying spontaneous movements (e.g., minimal limb movement or coughing) without performing a pulse check. “Checking for chest expansion” involved visual inspection of thoracic rise during simulated breaths, and participants were expected to both observe and verbalize their assessment. Although respiratory patterns in Rett syndrome may vary, chest expansion was assessed according to standard pediatric basic life support guidelines as an observable indicator of effective ventilation.

“Compression technique” was defined as maintaining correct hand placement, delivering vertical compressions using body weight with straight arms, and avoiding leaning to allow complete chest recoil. Rate, depth, and recoil quality were scored separately under dedicated checklist items. When a step could not be physically demonstrated on the mannequin, correct verbalization by the caregiver was accepted as evidence of appropriate assessment or intent. These standardized principles were applied consistently across all 16 checklist steps to ensure reliable scoring.

### Theoretical training

Participants received an overview of common respiratory and circulatory emergencies in children with Rett syndrome. This was followed by a review of the 2021 ERC PBLS guidelines and the basic principles of CPR, supported by video-enhanced content and live demonstrations using mannequins.

### Practical skills training

Following the theoretical session, participants practiced core PBLS skills individually under instructor supervision, including consciousness assessment, calling for help, cardiac arrest recognition, effective ventilation, and chest compressions. Each skill was repeated until competency was demonstrated, with continuous real-time feedback.

### Scenario-based training

After skill acquisition, participants completed a five-minute, single-rescuer PBLS simulation using medium-fidelity pediatric mannequins. Scenarios reflected realistic emergency situations and were video recorded with consent.

### Video-based debriefing

A structured debriefing was conducted immediately after simulation following the PEARLS framework (Promoting Excellence and Reflective Learning in Simulation). The session included:

1. Reaction: Participants shared initial emotional responses to facilitate psychological processing.
2. Analysis: Using an advocacy–inquiry approach, facilitators guided stepwise reflection on actions, clinical reasoning, and decision-making. Video playback was reviewed during group discussion to reinforce correct PBLS sequence execution and address performance gaps.
3. Summary: Key learning points were consolidated, and participants discussed application of PBLS skills in real-life caregiving situations.

Feedback focused on adherence to the PBLS algorithm, underlying clinical rationale, and achievement of predefined learning objectives.

### Post-training performance assessment

Participants repeated a five-minute PBLS simulation under identical assessment conditions using a different but clinically equivalent scenario. Two independent instructors evaluated performance using the same checklist and scoring criteria.

**Fig. 2:**
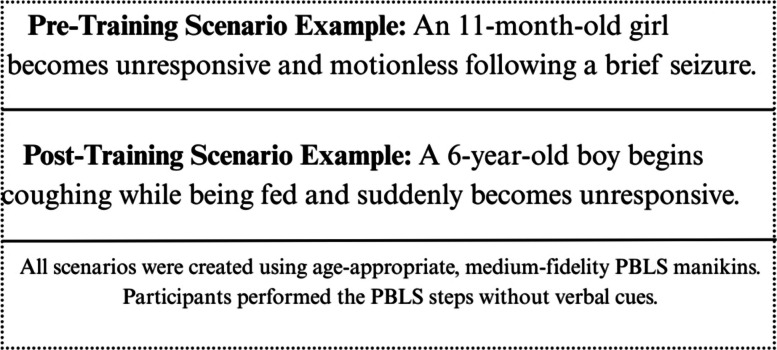
Training scenario descriptions

In both pre- and post-training assessments, participants managed an unresponsive, non-breathing child requiring full PBLS intervention. Contextual variations (e.g., collapse during feeding after coughing or following a brief seizure) were introduced to enhance realism while maintaining identical algorithmic requirements. Participants performed the sequence without verbal cues. Using equivalent but not identical scenarios minimized memorization and allowed for a more accurate assessment of true skill acquisition (Fig. 2).

### Training evaluation questionnaire

Following the simulation, participants completed a paper-based 13-item questionnaire assessing their satisfaction with the training. The questionnaire was adapted from validated international tools [9, 10] and included 5-point Likert-scale items (1 = strongly disagree, 5 = strongly agree) addressing six domains: (1) motivation and engagement, (2) training focus, (3) skill and confidence development, (4) training duration and content clarity, (5) effectiveness of teaching methods, and (6) teamwork and communication. The full questionnaire is provided in Additional file.

Descriptive statistics, including means and standard deviations, were used to summarize participants’ performance scores. The Shapiro–Wilk test was applied to assess the normality of data distribution. As the data did not meet the assumption of normality (*p* < 0.05), non-parametric tests were used. Specifically, the Wilcoxon Signed-Rank Test was employed to compare pre- and post-training performance scores. Performance was evaluated using a three-point scale: 10 points for correct execution, 5 points for partially correct execution, and 0 points for incorrect or omitted actions. All statistical analyses were performed using IBM SPSS Statistics for Windows, Version 23.0 (IBM Corp., Armonk, NY, USA). A *p*-value of less than 0.05 was considered statistically significant.

## Results

This single-group pre–post pilot study evaluated the impact of simulation-based PBLS training on caregiver performance. Demographic characteristics are summarized in Tables 1 and 2. None of the caregivers had prior PBLS training or a healthcare background; all were laypersons without clinical experience. The majority were female (70%) and aged 20–40 years (60%). None of the children had a previous cardiopulmonary arrest or CPR history.

**Table 1:** Details of the patient’s demographic data

Categories	n	%
Patient's Data		
Patient’s Gender		
*Male*	2	20
*Female*	8	80
Patient’s Age		
*1 month-1 year*	1	10
*2–6 years*	5	50
*6–18 years*	4	40
Epilepsy Diagnosis		
*Yes*	7	70
*No*	3	30
Chewing-Swallowing Problem		
*Yes*	2	20
*No*	8	80
Previous CPR Requirement		
*Yes*	0	0
*No*	10	100

**Table 2:** Details of the participant’s demographic data

Categories	n	%
Participant’s Data		
Participant’s Gender		
*Male*	3	30
*Female*	7	70
Participant’s Age		
*20–40 years*	6	60
*40–60 years*	4	40
PBLS Training Experience		
*Yes*	0	0
*No*	10	100

Wilcoxon Signed-Rank Tests indicated a statistically significant improvement in post-training performance scores across most PBLS steps. (Table 3).

**Table 3:** Detailed analysis of the PBLS checklist before and after training

**Check**l**ist Steps**	**Pre-Test’s Evaluation**				**Post-Test’s Evaluation**				***p*-value**	**Cohen’s d**
	**Successful Attempt (%)**	**Mean**	**Median**	**SD**	**Successful Attempt (%)**	**Mean**	**Median**	**SD**		
Check for consciousness	0	5	5	0	100	10	10	0	0.002	NaN
Shout for help	60	6	10	5.164	80	8	10	4.216	0.157	0.42
Check for breathing	0	3	5	2.582	80	8	10	4.216	0.007	1.43
Open airway	0	3.5	5	2.415	80	8	10	4.216	0.008	1.31
5-rescue breaths	0	0	0	0	80	8	10	4.216	0.005	2.68
Check for clear signs of life	80	8	10	4.216	100	10	10	0	0.157	0.67
Effective chest compressions										
*Hand position*	0	0	0	0	100	10	10	0	0.002	NaN
*Compression technique*	0	0	0	0	60	8	10	2.582	0.004	4.38
*Compression depth 4–5 cm*	0	0	0	0	60	8	10	2.582	0.004	4.38
*Compression rate 100–120/min*	0	0	0	0	60	8	10	2.582	0.004	4.38
*Check for recoil*	0	0	0	0	100	10	10	0	0.002	NaN
*Compression interval*	0	0	0	0	60	6	10	5.164	0.014	1.64
Effective ventilation										
*1 s inspiration*	0	0	0	0	90	9	10	3.162	0.003	4.03
*Check for chest expansion*	0	0	0	0	10	1	0	3.162	0.317	0.45
15 chest compressions, 2 rescue breaths	0	0	0	0	90	9	10	3.162	0.003	4.03
CPR for 1 min then call 112	0	0	0	0	60	6	10	5.164	0.014	1.64

**Fig. 3:**
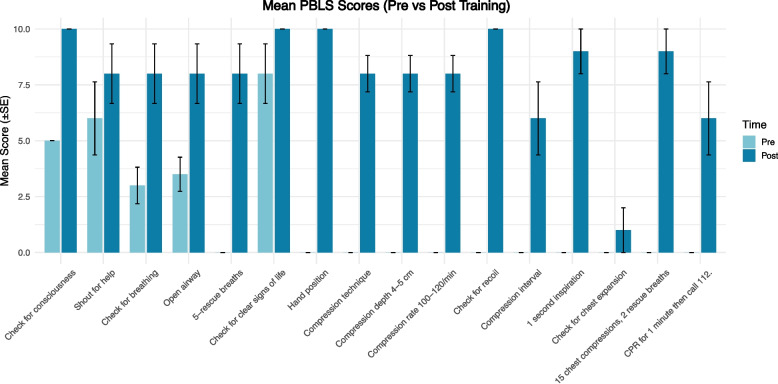
Visualization of the results: bar plot of mean scores with standard deviations

A comparison of PBLS performance before and after training is presented in Table 3. The median total score increased markedly from a pre-training mean of 25.5 (SD: ± 10.1) to a post-training mean of 127 (SD: ± 22.1), indicating a substantial improvement in participants’ PBLS skills. Figure 3 displays a visual summary of these changes.

Table 3 provides a detailed analysis of each step of the PBLS checklist before and after training.

The “consciousness check” (*p* = 0.002) and “breathing check” (*p* = 0.01) steps reached the highest possible scores after training. Significant improvement was observed in the “rescue breathing” step (*p* = 0.006), indicating better understanding and application of ventilation techniques. “Effective chest compressions” improved across all parameters (hand position, technique, compression depth, ratio), with *p* < 0.05 and Cohen’s d > 1, indicating notable development. “Effective ventilation” (*p* = 0.003) showed statistically significant improvement. Overall, simulation-based PBLS training had a statistically significant effect on participant performance (*p* < 0.05; ES > 0.4). Although certain steps, such as “calling for help” and “looking for clear signs of life,” did not reach statistical significance, post-training mean scores increased with a moderate effect size (Cohen’s d > 0.4), suggesting a potential training effect limited by the small sample size. Prior to the training, the success rate (defined as a score of 10) was low or nonexistent for most PBLS steps. After training, the proportion of successful attempts increased significantly across key actions such as effective ventilation and CPR sequence adherence.

Table 4 shows training assessment questionnaire results, where the mean scores exceeded 4.7 out of 5. Participants felt motivated and actively engaged in the simulation (mean: 4.77). They reported that the simulation enhanced focus and provided a structured learning environment (mean: 4.83). They gained confidence and developed skills (mean: 5). The training duration was well-utilized, and the content was clear (mean: 4.87). The simulation was effective in improving collaboration and communication skills (mean: 5).

**Table 4:** Simulation-based training assessment questionnaire results

Questions	Mean	SD
Participation and motivation	4.77	0.25
*Simulation training encouraged me to participate in the training*	4.5	0.850
*The simulation training encouraged me to be an active participant*	4.8	0.42
*As a participant, the simulation training motivated me*	5	0
Training focus and concentration	4.83	0.15
*Simulation training was participant-centered*	4.8	0.63
*During the simulation scenario training, the environment was education-focused*	4.7	0.48
*The simulation training increased concentration on the education*	5	0
Development of skills and confidence		
*Simulation training helped me improve my competency in PBLS*	5	0
*Simulation training helped me improve my self-confidence*	5	0
Training duration and content	4.87	0.06
*The simulation training time was used very effectively*	4.9	0.32
*With simulation training, understanding or interpreting the topic of PBLS was easy*	4.8	0.63
*I can use what I have learned about PBLS throughout my life*	4.9	0.32
Effectiveness of methods and strategies		
*The teaching methods used in this simulation were helpful and effective*	4.7	0.48
Collaboration and communication		
*Simulation training provided a structured social learning environment*	5	0

## Discussion

This pilot study is among the first to assess the impact of simulation-based PBLS training for caregivers of children with Rett syndrome. To our knowledge, it also represents the first initiative in Turkey to repurpose university-based medical simulation centers for a public health objective.

The findings of our study suggest that simulation-based training can enhance caregivers’ ability to perform key steps of the PBLS algorithm. Caregivers demonstrated limited baseline proficiency, which is consistent with previous reports showing low pre-training resuscitation skills among laypersons. After the training, statistically significant improvements were observed across multiple PBLS components (*p* < 0.05), particularly in airway opening, rescue breathing, and chest compression quality, suggesting that a brief, structured simulation session may contribute to the acquisition of essential life-support skills. Questionnaire findings also showed increased confidence and positive perceptions of the training experience, suggesting that simulation-based training may help caregivers feel better prepared to respond to emergencies.

Timely and effective pre-hospital intervention improves survival and neurological outcomes in pediatric cardiac arrest [17]. Despite this, bystander-initiated CPR rates remain low, particularly in children [18]. This gap is especially concerning for children with neurodevelopmental disorders such as Rett syndrome, who may experience acute cardiopulmonary events or severe seizures [19].

Caregiver CPR training has been shown to increase confidence, improve response accuracy, and reduce delays in resuscitation initiation. Accordingly, data from large pediatric cardiac arrest cohorts support the value of strengthening bystander PBLS skills as a practical strategy to improve emergency preparedness among caregivers of medically vulnerable children [17].

Simulation-based education is widely used in healthcare training to bridge the gap between theoretical knowledge and practical skills [20]. By providing a safe and controlled learning environment, it enables hands-on practice without patient risk, reinforcing competence and confidence [21, 22].

Consistent with our findings, prior studies have demonstrated that simulation-based training enhances preparedness among caregivers of high-risk pediatric populations [23, 24]. A recent meta-analysis further reported improved quality of care delivered by parents of chronically ill children following simulation training [22], and simulation-based BLS instruction before NICU discharge has been shown to significantly improve parental performance [14].

Looking ahead, our long-term aim is to extend similar simulation-based training initiatives to caregivers of children with other high-risk conditions associated with sudden deterioration. Although such training cannot prevent all medical complications, improving caregivers’ ability to recognize early warning signs and initiate appropriate first-aid responses may enhance preparedness and reduce delays in emergency situations. In this context, simulation centers may serve as valuable community-based resources that support caregiver education and strengthen emergency preparedness beyond the hospital setting.

### Strengths and limitations

A key strength of this study is the structured and objective design of the training module, which was aligned with clearly defined learning objectives and standardized assessment criteria. The program effectively reduced baseline performance variability and promoted measurable skill acquisition in PBLS.

Several limitations should be acknowledged. First, the small sample size (*n* = 10) limits generalizability. Although appropriate for a feasibility-focused pilot study, larger studies are needed to confirm these findings.

Second, Rett syndrome is clinically heterogeneous. Detailed phenotypic severity data weren’t collected, limiting our ability to assess how disease severity may have influenced caregiver performance. The inclusion of two male participants—who often present with more severe phenotypes in X-linked conditions—may reflect a more clinically affected subgroup and potentially higher caregiver motivation.

Third, all training and assessments were conducted in a simulation center. While this ensured standardization, it may not fully reflect real-life home or community emergencies and therefore may limit conclusions regarding long-term behavioural transfer.

Fourth, only PBLS psychomotor performance was formally evaluated. Although seizure first aid and choking management were included in the training, these components were not objectively assessed. Future studies should incorporate broader and multi-domain outcome measures.

Finally, the study assessed short-term outcomes only and was conducted at a single center. Longitudinal follow-up and multi-center studies are required to evaluate skill retention, external validity, and broader public health applicability.

## Conclusion

This pilot study demonstrates that simulation-based PBLS training is an effective and feasible method for educating caregivers of children with Rett syndrome. The training significantly improved participants’ emergency response performance enhanced their confidence, and provided a structured, engaging learning experience.

Given the high risk of sudden life-threatening events in children with neurodevelopmental disorders, caregiver training in PBLS is essential. The repurposing of university-based medical simulation centers for public health-oriented education, as illustrated in this study, represents a novel and impactful approach to community engagement.

To sustain and further extend the benefits of such training, regular refresher sessions and follow-up programs are recommended. Periodic reassessment may help ensure the retention of critical skills and maintain caregiver readiness.

Future research should aim to evaluate the long-term impact of simulation-based PBLS training on both caregiver performance and patient outcomes, ideally through studies with larger sample sizes and longitudinal designs. Additionally, expanding such training initiatives to include caregivers of other high-risk pediatric populations may help address broader gaps in community-based emergency preparedness.

## Supplementary Information

Supplementary Material 1

## Data Availability

The datasets used and/or analysed during the current study are available from the corresponding author on reasonable request.

## Abbreviations

PBLS: Pediatric basic life support
ERC: European Resuscitation Council
*MECP2*: Methyl-CpG-binding protein 2
SUDEP: Sudden unexpected death in epilepsy
BLS: Basic Life Support
NICU: Neonatal intensive care unit
CPR: Cardiopulmonary resuscitation
PEARLS: Promoting Excellence and Reflective Learning in Simulation
CASE: Centre of Advanced Simulation and Education
ACURARE: Acıbadem University Rare Diseases& Orphan Drugs Application& Research Center
