# Supplementary material for: Pilot study: the impact of simulation-based pediatric basic life support training on the performance of caregivers of children with Rett syndrome
Source: BMC Med Educ. 2026 Apr 10;26:836. doi: 10.1186/s12909-026-09158-y (PMC13217667; doi:10.1186/s12909-026-09158-y)
Supplement: Supplementary file 1 — Supplementary Material 1 [file 12909_2026_9158_MOESM1_ESM.docx]

# Simulation-Based Learning Evaluation Survey (5-Point Likert Scale)

Please indicate your level of agreement with each statement by marking the appropriate box.
1 = Strongly Disagree 2 = Disagree 3 = Neutral 4 = Agree 5 = Strongly Agree

|  | STATEMENT | 1 | 2 | 3 | 4 | 5 |
| --- | --- | --- | --- | --- | --- | --- |
|  | **Participation and motivation** |  |  |  |  |  |
| 1 | *Simulation training encouraged me to participate in the training* |  |  |  |  |  |
| 2 | *The simulation training encouraged me to be an active participant* |  |  |  |  |  |
| 3 | *As a participant, the simulation training motivated me* |  |  |  |  |  |
|  | Training focus and concentration |  |  |  |  |  |
| 4 | *Simulation training was participant-centered* |  |  |  |  |  |
| 6 | *During the simulation scenario training, the environment was education-focused* |  |  |  |  |  |
| 6 | *The simulation training increased concentration on the education* |  |  |  |  |  |
|  | Development of skills and confidence |  |  |  |  |  |
| 7 | *Simulation training helped me improve my competency in PBLS* |  |  |  |  |  |
| 8 | *Simulation training helped me improve my self-confidence* |  |  |  |  |  |
|  | Training duration and content |  |  |  |  |  |
| 9 | *The simulation training time was used very effectively* |  |  |  |  |  |
| 10 | *With simulation training, understanding or interpreting the topic of PBLS was easy* |  |  |  |  |  |
| 11 | *I can use what I have learned about PBLS throughout my life* |  |  |  |  |  |
|  | Effectiveness of methods and strategies |  |  |  |  |  |
| 12 | *The teaching methods used in this simulation were helpful and effective* |  |  |  |  |  |
|  | Collaboration and communication |  |  |  |  |  |
| 13 | *Simulation training provided a structured social learning environment* |  |  |  |  |  |
